# *Pseudomonas aeruginosa* detachment from surfaces *via* a self-made small molecule

**DOI:** 10.1016/j.jbc.2021.100279

**Published:** 2021-01-12

**Authors:** Robert J. Scheffler, Yuki Sugimoto, Benjamin P. Bratton, Courtney K. Ellison, Matthias D. Koch, Mohamed S. Donia, Zemer Gitai

**Affiliations:** 1 Department of Molecular Biology, Princeton University, Princeton, New Jersey, USA; 2 Lewis-Sigler Institute for Integrative Genomics, Princeton University, Princeton, New Jersey, USA

**Keywords:** *Pseudomonas aeruginosa*, surface detachment, bioactivity-guided fractionation, natural product, type IV pili, bacterial adhesion, microbiology, HAI, hospital-acquired infection, HHQ, 2-heptyl-4-hydroxyquinoline, HPLC, high-performance liquid chromatography, HPLC-MS, HPLC coupled with mass spectrometry, HPLC-HRMS, HPLC high-resolution MS, LB, Luria–Bertani, MHQ, 2-methyl-4-hydroxyquinoline, NMR, nuclear magnetic resonance, PBS, phosphate buffered saline, PQS, *Pseudomonas* quinolone signaling, TFP, type IV pili

## Abstract

*Pseudomonas aeruginosa* is a significant threat in both healthcare and industrial biofouling. Surface attachment of *P. aeruginosa* is particularly problematic as surface association induces virulence and is necessary for the ensuing process of biofilm formation, which hampers antibiotic treatments. Previous efforts have searched for dispersal agents of mature biofilm collectives, but there are no known factors that specifically disperse individual surface-attached *P. aeruginosa*. In this study, we develop a quantitative single-cell surface-dispersal assay and use it to show that *P. aeruginosa* itself produces factors that can stimulate its dispersal. Through bioactivity-guided fractionation, mass spectrometry, and nuclear magnetic resonance, we elucidated the structure of one such factor, 2-methyl-4-hydroxyquinoline (MHQ). MHQ is an alkyl quinolone with a previously unknown activity and is synthesized by the PqsABC enzymes. Pure MHQ is sufficient to disperse *P. aeruginosa,* but the dispersal activity of natural *P. aeruginosa* conditioned media requires additional factors. Whereas other alkyl quinolones have been shown to act as antibiotics or membrane depolarizers, MHQ lacks these activities and known antibiotics do not induce dispersal. In contrast, we show that MHQ inhibits the activity of Type IV Pili (TFP) and that TFP targeting can explain its dispersal activity. Our work thus identifies single-cell surface dispersal as a new activity of *P. aeruginosa-*produced small molecules, characterizes MHQ as a promising dispersal agent, and establishes TFP inhibition as a viable mechanism for *P. aeruginosa* dispersal.

Hospital-acquired infections (HAIs) and the pathogens that cause them are a growing concern due to the rise in antibiotic resistance, which makes treating these infections increasingly difficult (1). Multi-drug-resistant pathogens are particularly problematic in healthcare settings as hospital patients are often immunocompromised and contaminated surfaces promote bacterial transfer to new patients. The list of contaminated surfaces ranges from medical implants to neckties worn by doctors.

One of the major causes of a wide variety of HAIs is the bacterium *Pseudomonas aeruginosa* (1, 2). *P. aeruginosa* produces a number of secreted factors including small molecules such as pyocyanin (3), glycolipids such as rhamnolipids (4), secreted proteins such as elastase (5), and nucleic acids (6). These various factors allow *P. aeruginosa* to inhabit a wide range of environments and infect a surprising array of hosts (7, 8). Recently, our lab and others demonstrated that intital surface attachment by individual cells strongly induces *P. aeruginosa* virulence (9, 10, 11, 12). To initiate surface-induced virulence, *P. aeruginosa* senses surfaces through type IV pili (TFP), which are extracellular polymers that can be actively extended and retracted (10, 13). Thus, disrupting surface attachment or TFP activity could be powerful yet largely untapped methods to combat *P. aeruginosa* pathogenesis, by reducing its propensity to disseminate *via* surfaces and by reducing the induction of its virulence mechanisms.

We sought to identify new ways of disrupting single-cell surface attachment through chemical means such as small molecules. *P*. *aeruginosa* produces many secreted metabolites with a wide array of functions (14). These secreted factors include virulence factors such as pyocyanin and rhamnolipids, as well as factors that regulate specific aspects of the *P. aeruginosa* life cycle like the biofilm dispersal cue, *cis*-2-decenoic acid (15). A number of groups have screened for compounds that disrupt mature multicellular biofilms (15, 16, 17, 18), but significantly less work has been done on inhibitors of early surface attachment by single cells.

Here we show that cell-free supernatant from cultures of *P. aeruginosa* PA14 causes rapid dispersal of cells from the surface. Using a bioactivity-guided chemical fractionation and purification approach, we identify 2-methyl-4-hydroxyquinoline (MHQ) as a small molecule factor made by *P. aeruginosa* that induces its own dispersal. MHQ has not been previously characterized with respect to its function or synthesis. We show that MHQ is synthesized by enzymes in the *Pseudomonas* quinolone signaling (PQS) pathway. Furthermore, we find that MHQ inhibits TFP pilus activity, potentially explaining how MHQ causes dispersal of *P. aeruginosa* from the surface. Our findings thus characterize a previously understudied secreted metabolite with the potential to prevent *P. aeruginosa* infections by limiting initial surface attachment.

## Results

### P. aeruginosa conditioned media removes P. aeruginosa from a surface

**Figure 1:**
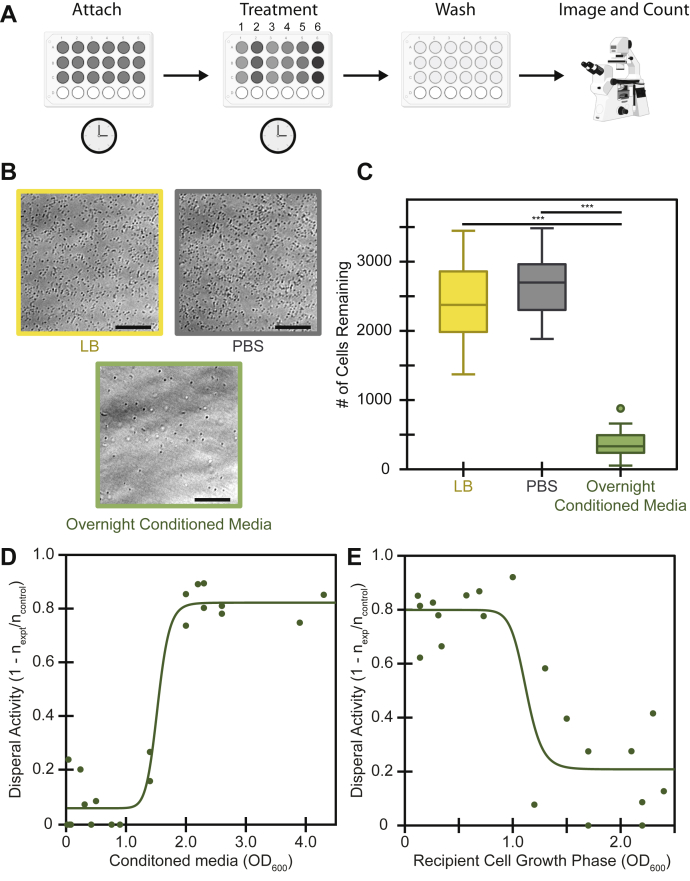
**Conditioned media from *P. aeruginosa* disperses *P. aeruginosa* cells**. *A*, schematic of the DISPEL assay. Dispersal of initially surface-attached pathogens via extract lavage (DISPEL) involves the following: attaching cells for 10 min; removing planktonic culture; treating attached cells for 10 min; gently washing to remove treatment and detached cells; imaging and counting remaining attached single cells. *B* and *C*, control treatments of LB or PBS are compared with experimental treatments such as cell-free media from an overnight culture. *B*, example phase-contrast micrographs (scale bar 40 μm). *C*, quantification of micrographs depicted in box and whisker plots from 24 biological replicates. ∗∗∗ *p*-value < 0.001 from Student's *t*-test. *D* and *E*, dispersal activity is bounded between 0 (no cells removed) and 1 (all cells removed) and is calculated as 1 – (cells still attached post experimental treatment/cells still attached post control treatment). Fits are based on modified Hill equation (Equation 2). *D*, dispersal activity of cell-free conditioned media from varied OD_600_ cultures on mid-log (OD_600_ 0.6–0.8) *P. aeruginosa* cells. *E*, dispersal activity of cell-free overnight conditioned media on *P. aeruginosa* cells from varied OD_600_ cultures. Before attachment, cultures were diluted or concentrated to standardize the seeding density.

Since *P. aeruginosa* is known to produce a staggering array of secreted secondary metabolites with a variety of biological functions, we investigated whether it might produce a compound that would cause cells to detach from a surface. To test a variety of conditions in a high-throughput manner, we designed an assay we term DISPEL, for dispersal of initially surface-attached pathogens *via* extract lavage. In brief, *P. aeruginosa* cells were first allowed to attach to the surface of a 96-well plate, the unattached cells were removed by washing, the attached cells were treated with molecules or supernatants of interest, the cells were then washed to remove dispersed cells, and the plate was imaged to determine the number of surface-attached cells remaining (Fig. 1, *A* and *B*). Figure 1*C* shows a quantification of the number of mid-log recipient cells that remained attached after treatment with phosphate buffered saline (PBS), Luria–Bertani medium (LB), or cell-free supernatant from an overnight culture.

To determine when *P. aeruginosa* produces a dispersal signal, we used the DISPEL assay to test the dispersal activity of cells from different growth phases. We therefore tested cell-free supernatant from *P. aeruginosa* cultures grown to different densities for their ability to disperse mid-log *P. aeruginosa* (OD = 0.6–0.8) (Fig. 1*D*). We defined dispersal activity as the fraction of cells removed by the treatment (1 – n_(cells remaining after treatment)_/n_(cells remaining after control)_). As controls, cells treated with either LB growth medium or PBS remained attached to the surface in large numbers with little to no dispersal activity relative to one another (Fig. 1, *B* and *C*). In contrast, cells treated with cell-free overnight conditioned media and with supernatants from cultures of OD_600_ greater than 1.5 were largely removed from the surface (Fig. 1*D*). Conditioned media from cultures at OD_600_ less than 1.5 showed little to no activity (Fig. 1*D*).

Because dispersal activity of supernatant is dependent on the culture growth phase, we also sought to determine whether dispersal response of the attached cells is growth phase specific. To test this, we grew *P. aeruginosa* over a range of cell densities, normalized their cell numbers, and allowed those cells to attach to the surface. We then treated each sample with conditioned media from late stationary growth-phase cultures of *P. aeruginosa* and quantified the extent of dispersal. Recipient cells that were below an OD_600_ = 1.0 showed high dispersal, whereas cells above OD_600_ = 1.0 showed low dispersal (Fig. 1*E*). These data show that as *P. aeruginosa* cultures become denser, their supernatants increase in dispersal activity, but the cells themselves become less responsive to the dispersal cue, suggesting a mechanism for growth-phase-specific dispersal and attachment. To further study this phenomenon, we thus focused on overnight-grown signalers and mid-log recipients.

### Bioactivity-guided fractionation identifies MHQ as a P. aeruginosa dispersal agent

**Figure 2:**
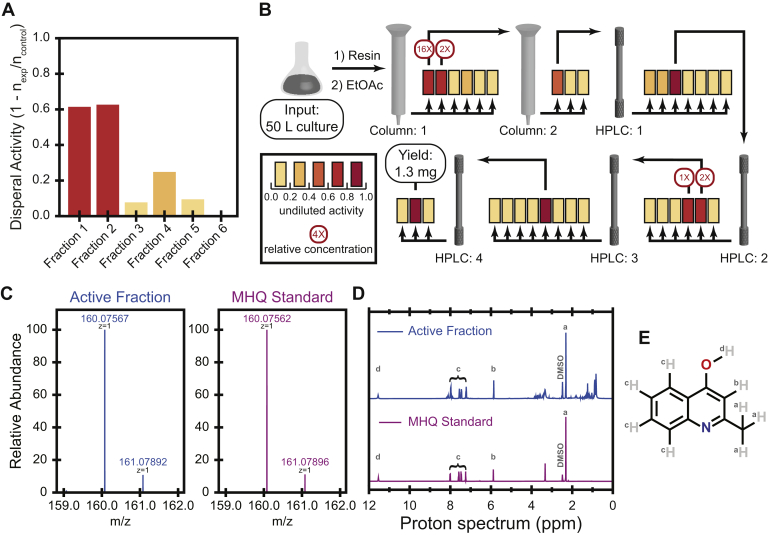
**Bioactivity-guided fractionation identified MHQ as a *P. aeruginosa* dispersal agent**. *A*, fractionation of crude extract from 50 L of overnight conditioned media of *P. aeruginosa* revealed that the first two fractions contained dispersal activity against mid-log (OD_600_ 0.6–0.8) *P. aeruginosa* cells. *B*, we utilized our DISPEL assay to perform bioactivity-guided fractionation. Multiple rounds of fractionation continued to retain activity until the sample was pure enough for further chemical analysis. *C*, high-resolution mass spectrum of the purified MHQ in the final active fraction (VI). *D*, ^1^H-NMR spectrum of purified MHQ in the final active fraction VI in comparison with that of an authentic standard of the same molecule obtained commercially. See also Figures S2–S4 and Table 1 for additional NMR spectra and peak assignments. (E) MHQ is an alkyl quinolone with a single methyl as the tail group.

To characterize the chemical nature of the dispersal activity in *P. aeruginosa* supernatants, we first used a variety of perturbations. These results suggested that the activity is mediated by a small organic molecule as the activity was protease and nuclease-insensitive, heat-stable, and <5 kDa in size. Consequently, we generated 50 L of PA14 cell-free supernatant from overnight cultures and isolated the small molecules using Diaion HP-20 resin followed by ethyl acetate extraction. This crude extract was fractionated on an open Mega Bond Elut-C18 column. Two of these fractions retained significant dispersal activity, confirming the initial indications that the activity is mediated by a small molecule (Fig. 2*A*).

To determine which fraction had a higher concentration of active molecule, we assayed serial dilutions of the active fractions and determined that the first fraction (I) had approximately eightfold higher concentration of active molecule. We thus subfractionated I using a second open Mega Bond Elut-C18 column and identified a single subfraction (II) that retained dispersal activity. II was further subfractionated using four subsequent rounds of high-performance liquid chromatography (HPLC) C-18 column fractionation (we designated the subfraction with the highest activity in each round with roman numerals III–VI). Activity of the extract was assessed after each round of fractionation to ensure activity was not lost throughout the process (Fig. 2*B*).

The final round of fractionation yielded 1.3 mg of total material (VI) that was active in the dispersal assay. HPLC coupled with mass spectrometry (HPLC-MS) and HPLC high-resolution MS (HPLC-HRMS) analyses of this final subfraction indicated that it was dominated by a single molecular species with a mass-to-charge ratio (m/z) of 160.076 [M+H]^+^ (Fig. 2*C*), which corresponds to a molecule with a predicted mass of 159.068. From this mass we determined the most likely chemical formula of the dominant species as C_10_H_9_NO. To determine its structure, we performed nuclear magnetic resonance (NMR) analysis on VI, which revealed the dominant molecule's structure to be 2-methyl-4-hydroxyquinoline (MHQ) (Fig. 2, *D* and *E*, Figs. S1–S3, Table 1). Finally, we obtained a commercial standard of MHQ (Sigma-Aldrich) and confirmed that it matched the active fraction exactly with respect to its retention time, MS, and NMR, thereby validating the structural elucidation (Fig. 2, *C* and *D*, Figs. S1–S4, Table 1). Alkyl quinolones can be found in either enol or keto forms (19). Our structural elucidation indicated that VI and MHQ were both in the enol form with the furthest downfield proton corresponding to the hydroxyl proton at 11.6 ppm (Figs. S1–S3, Table 1).

**Table 1:** NMR peak assignments

No.	Final active fraction (VI)		MHQ standard		Pseudan I^a^ (30)
	^1^H δ (*J*, Hz) (500.40 MHz)	^13^C δ (125.84 MHz)	^1^H δ (*J*, Hz) (500.40 MHz)	^13^C δ (125.84 MHz)	^1^H δ (*J*, Hz)
2		149.7		149.6	
3	5.90 (s)	108.4	5.90 (s)	108.4	6.01 (s)
4		176.8		176.7	
	11.62 (s, OH)		11.56 (s, OH)		11.35 (br s, OH)
4a		124.5		124.5	
5	8.02 (d, 9.5)	124.8	8.02 (d, 9.6)	124.8	8.16 (d)
6	7.26 (t, 7.5)	122.7	7.26 (t, 8.1)	122.7	7.21 (t)
7	7.60 (t, 8.4)	131.5	7.60 (t, 8.4)	131.5	7.53 (m)
8	7.49 (d, 8.3)	117.8	7.48 (d, 8.2)	117.7	7.48 (m)
8a		140.1		140.1	
9	2.33 (s)	19.5	2.33	19.5	2.34 (s)

a Pseudan I was the name given to MHQ in (30).

**Figure 3:**
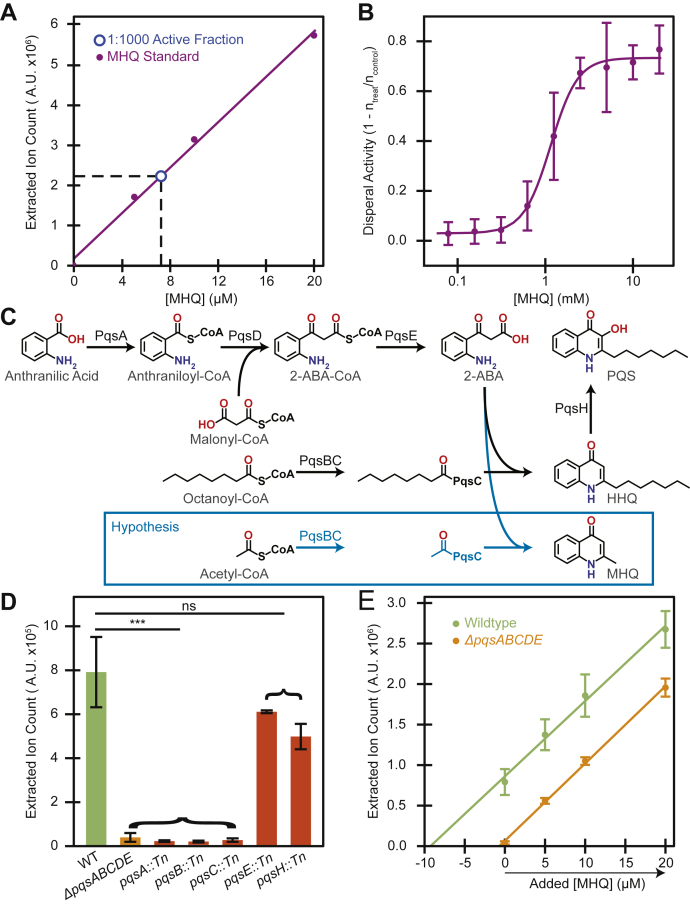
**Potency and biosynthesis of MHQ**. *A*, quantification of MHQ concentration in VI (by HPLC-MS) using commercially available MHQ as a standard. *B*, concentration-dependent dispersal activity of MHQ on mid-log (OD_600_ 0.6–0.8) *P. aeruginosa* cells. Mean and standard deviation shown from four biological replicates. Fit is based on modified Hill equation (Equation 2). *C*, schematic of the HHQ biosynthetic pathway. Due to the similarity of MHQ to HHQ, we hypothesized that it is produced by the same pathway. For consistency with *P*QS literature MHQ is shown in the keto form. *D*, relative concentration of MHQ in various conditioned media. Mean and standard deviation shown from five biological replicates (WT and *ΔpqsABCDE*) or two biological replicates (transposon disruptions). ns *p*-value > 0.05; ∗∗∗ *p*-value < 0.001 from Student's *t*-test compared with WT conditioned media. *E*, an absolute concentration measurement of MHQ in conditioned media (by HPLC-MS) using the standard addition method. Mean and standard deviation shown from five biological replicates.

To determine if MHQ indeed has dispersal activity, as predicted by the bioactivity-guided fractionation, we tested the activity of the commercial MHQ standard at different concentrations using the DISPEL assay. HPLC-MS indicated that the final active subfraction (VI) contained roughly 7.3 mM MHQ (Fig. 3*A*). Purified MHQ was capable of dispersing *P. aeruginosa* at similar levels (Fig. 3*B*). A dilution series of MHQ revealed that its effective concentration for dispersal activity (EC_50_) is roughly 1 mM (Fig. 3*B*). Together these data confirmed that MHQ is both made by *P. aeruginosa* and capable of dispersing these bacteria from a surface.

### MHQ is synthesized by the PQS pathway

MHQ is an alkyl quinolone and resembles 2-heptyl-4-hydroxyquinoline (HHQ) with the heptyl tail replaced by a single methyl tail (Fig. 3*C*). Since HHQ and other alkyl quinolones are synthesized by the enzymes encoded by the *pqsABCDE* operon, we hypothesized that these enzymes may also be responsible for the production of MHQ (Fig. 3*C*). Specifically, if PqsBC uses acetyl-CoA as a substrate instead of octanoyl-CoA, this would convert the HHQ precursor, 2-ABA, into MHQ (Fig. 3*C*). To test this hypothesis, we deleted the entire *pqsABCDE* operon and compared the relative amount of MHQ in supernatants from this operon deletion with that of WT (see Experimental procedures for details). We found that the operon mutant eliminated the HPLC-MS peak associated with MHQ, indicating that these genes are indeed required for producing MHQ (Fig. 3*D*). To attempt to assess the roles of individual genes within the operon, we used mutants containing transposon insertions in each gene (20). We found that interrupting *pqsA*, *pqsB*, or *pqsC* caused a significant decrease in the amount of MHQ produced, while disruption of *pqsE* or *pqsH* did not have a significant impact on MHQ levels (Fig. 3*D*). These results are consistent with our hypothesis that MHQ production requires the PqsA, PqsB, and PqsC enzymes. Based on its chemical structure and similarity to HHQ, we predicted that PqsE would also be required for MHQ synthesis. However, a recent report suggested that another enzyme (TesB) has redundant activity with PqsE in *P. aeruginosa,* which may explain why MHQ is still produced in this mutant (21).

Since MHQ is produced by the *pqsABCDE* operon, we determined if other alkyl quinolones also dispersed cells from the surface. Treatment of wild-type PA14 in the DISPEL assay with alkyl quinolones of various tail lengths longer than MHQ, including HHQ, all resulted in no dispersal (Fig. S6*A*). Furthermore, many alkyl quinolone activities function by inducing gene expression through activation of the PqsR transcriptional regulator. In contrast, *ΔpqsR* mutants were still dispersed by MHQ, and MHQ still dispersed *P. aeruginosa* whose new protein synthesis was inhibited by gentamycin cotreatment (Fig. S6, *B* and *C*). These results suggest that among alkyl quinolone, dispersal activity is specific for MHQ, and that MHQ's dispersal activity is independent of PqsR and the induction of new protein synthesis.

### Conditioned media contains dilute concentrations of MHQ

Relative concentration measurements were sufficient to establish that the PQS enzymes are required for MHQ synthesis but could not address the absolute levels of MHQ found in conditioned media. Given the complex mixture of molecules in conditioned media that could affect ionization efficiency, we used the standard addition approach to quantify absolute MHQ concentrations. Briefly, we added known concentrations of MHQ to the conditioned media and used HPLC-MS to generate a calibration curve. Extrapolating this calibration curve back to the X-intercept established the absolute concentration of MHQ that would be present with no additional standard. Using this approach, we found that WT conditioned media contained roughly 10 μM MHQ (Fig. 3*E*). Conditioned media from the *ΔpqsABCDE* mutant contained less than 1 μM MHQ, consistent with our relative concentration measurements (Fig. 3*E*). Since the EC_50_ of MHQ for *P. aeruginosa* dispersal is roughly 1 mM (Fig. 3*B*), the significantly lower concentration of MHQ in the WT conditioned media indicates that either MHQ activity is strongly potentiated in WT conditioned media or there are additional factors in WT conditioned media that lead to its dispersal activity.

To directly determine if MHQ is required for WT dispersal activity, we tested the dispersal activity of conditioned media from the *ΔpqsABCDE* mutant that lacks detectable MHQ. Conditioned media from the *ΔpqsABCDE* mutant retained full activity in the DISPEL assay, supporting the conclusion that the WT dispersal activity does not require MHQ (Fig. S5). In addition to MHQ, the PQS pathway produces a wide range of small molecules with downstream effects on quorum sensing, virulence, and secondary metabolism (22). Thus, the full activity from the conditioned media of the *ΔpqsABCDE* mutant indicates that the MHQ-independent dispersal factors in conditioned media are not the product of the PQS pathway or its downstream signaling.

### MHQ dispersal is not due to antibiotic activity or membrane depolarization

**Figure 4:**
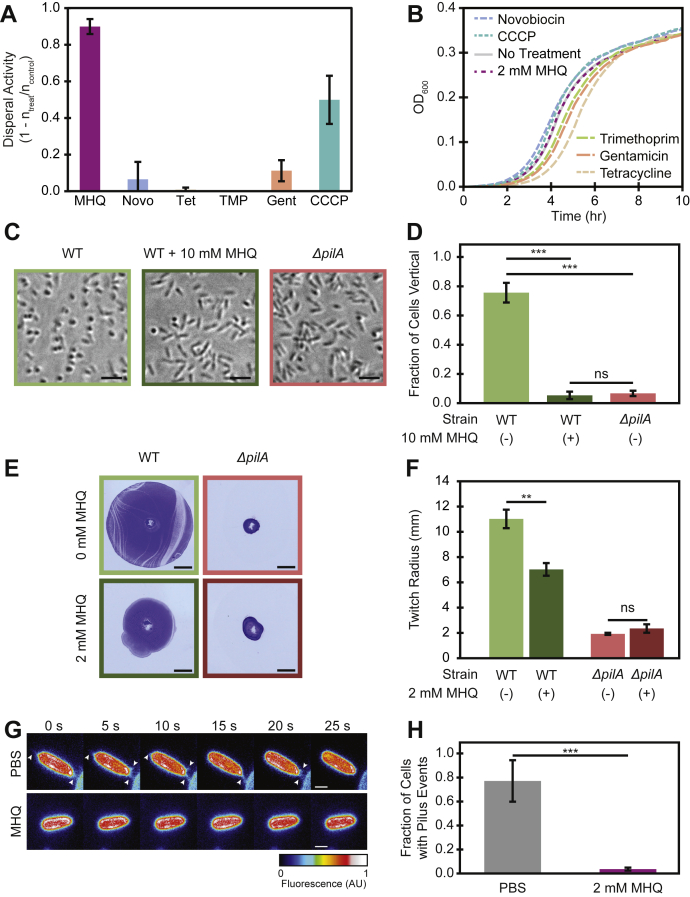
**MHQ inhibits Type IV pilus (TFP) activity**. *A*, known antibiotics, other than CCCP, do not cause dispersal activity in the DISPEL assay against mid-log (OD_600_ 0.6–0.8) *P. aeruginosa* cells. Mean and standard deviation shown from three biological replicates. Novobiocin—10 mg/ml, Tetracycline—16 μg/ml, Trimethoprim—125 μg/ml, Gentamicin—6 μg/ml, CCCP—200 μM, MHQ—2 mM. *B*, growth curve of *P. aeruginosa* after 10 min treatment with MHQ or known antibiotics. Mean shown for three biological replicates. *C* and *D*, high-magnification phase-contrast imaging of wildtype *P. aeruginosa* after treatment with PBS (n_total_ = 1347) or MHQ (n_total_ = 1331) for 5 min and *ΔpilA* cells treated with PBS (n_total_ = 1995). *C,* example micrograph. Scale bar 5 μm. *D*, fraction of cells per image that were aligned vertical to the surface. Mean and standard deviation shown from three biological replicates. ns *p*-value > 0.05; ∗∗∗ *p*-value < 0.001 from Student's *t*-test. *E* and *F*, TFP-dependent twitching assay in the presence or absence of MHQ. *E*, example twitch rings. Scale bar 5 mm. *F*, mean and standard deviation shown from three biological replicates. ns *p*-value > 0.05; ∗∗ *p*-value < 0.01 from Student's *t*-test. *G* and *H*, microscopic observation of pilus activity treated with PBS (n_total_ = 473) or 2 mM MHQ (n_total_ = 493). *G*, snapshot montage of *P. aeruginosa* cells with labeled pili. *Arrowheads* indicate extending or retracting pili. Scale bar 1 μm. *H*, fraction of cells per image that had at least one pilus event. Mean and standard deviation shown from three biological replicates. ∗∗∗ *p*-value < 0.001 from Student's *t*-test.

Despite the fact that MHQ is not required for WT conditioned media dispersal activity, MHQ possesses activity on its own and thus could prove to be a useful dispersal agent. Consequently, we sought to determine the mechanism by which MHQ causes dispersal, to both better characterize MHQ and determine how additional dispersal factors might function. MHQ chemically resembles other alkyl quinolones that have been associated with a wide range of biological functions including antibiotic activity and membrane depolarization (22, 23, 24). In our assay, 10 min of treatment with MHQ is sufficient to induce dispersal. To determine the feasibility of the dispersal being due to antibiotic activity, we sought to determine if a 10 min treatment with known antibiotics also induces dispersal. Thus, we used the DISPEL assay to compare the effect of MHQ treatment with treatment with known antibiotics of varying mechanisms of action: novobiocin (replication inhibitor), tetracycline (translation inhibitor), trimethoprim (metabolism disruptor), CCCP (membrane polarity disruptor), and gentamicin (translation inhibitor). None of these treatments resulted in as much dispersal as MHQ treatment, and only CCCP produced any significant dispersal activity (Fig. 4*A*). Furthermore, we confirmed that most of the antibiotics tested affected cell numbers after a 10 min treatment comparable with that used in the DISPEL assay, while MHQ had no effect on cell numbers in this time period (Figs. 4*B* and S6). These results suggest that antibiotic activity is insufficient to cause the dispersal of *P. aeruginosa* and that MHQ does not function as an antibiotic in the dispersal assay.

Since CCCP was the only antibiotic that caused even moderate dispersal (though still less than MHQ), and CCCP acts to depolarize bacterial membranes, we sought to determine if MHQ also perturbs membrane integrity. As a quantitative measure of membrane integrity, we used a flow cytometry assay utilizing TO-PRO3, which only stains cells that have been permeabilized, and DiOC_2_ (3), which shifts its emission wavelength based on membrane polarization. As positive controls we confirmed that polymyxin-B treatment led to the expected permeabilization of *P. aeruginosa* and CCCP treatment led to the expected depolarization of *E. coli lptD4213* (Fig. S7). We note that we were not able to use DiOC_2_ (3) staining in *P. aeruginosa* due to its inability to penetrate the outer membrane, whose integrity is compromised in *E. coli lptD4213*. *P. aeruginosa* cells treated with MHQ did not show an increase in TO-PRO3 staining, and *E. coli lptD4213* cells treated with MHQ did not show an increase in DiOC_2_ (3) staining. These results indicate that MHQ's mechanism of action is not mediated by membrane permeabilization or depolarization.

### MHQ inhibits type IV pilus activity

Since MHQ affects the association of *P. aeruginosa* cells with surfaces, we further investigated its potential mechanism of action by using high-magnification imaging to examine the behavior of individual *P. aeruginosa* cells at the surface. *P. aeruginosa* cells can attach to the surface either by their pole (vertically) or by their side (horizontally) (25, 26, 27). Upon treatment with PBS, 76% (n_total_ = 1347) of the cells attached to the surface vertically. In contrast, upon treatment with 10 mM MHQ, only 5% (n_total_ = 1331) of the cells attached to the surface vertically (Fig. 4, *C* and *D*).

We noticed a similar behavior between MHQ-treated WT cells and mutants lacking Type IV pilin subunits (PilA), as only 7% (n_total_ = 1995) of *ΔpilA* cells were attached vertically even in PBS treatment (Fig. 4, *C* and *D*). This result suggested that MHQ might disrupt type IV pilus (TFP) activity. Since TFP is required for twitching motility, we further characterized MHQ's effect on *P. aeruginosa* in a twitching assay (Fig. 4*E*). In this assay, cells are placed underneath agar and allowed to spread from their starting spot along the bottom surface of a Petri dish. The extent of this spread is visualized with crystal violet staining and quantified. In the presence of agar made with LB, WT cells traveled 11 ± 0.7 mm (mean ± SD) from the starting spot. In contrast, in the presence of agar made with LB and 2 mM MHQ, WT cells traveled significantly less (7 ± 0.5 mm, Fig. 4*F*). The effect of MHQ on twitching depended on TFP, as *ΔpilA* cells that lack TFP traveled similar distances away from the starting spot in the absence of MHQ (1.9 ± 0.1 mm) or in the presence of MHQ (2.3 ± 0.3 mm) (Fig. 4*F*).

To directly assay the effect of MHQ on TFP activity, we used the recently developed cysteine-labeling approach to fluorescently label TFP and image their dynamics (13, 28, 29). This approach uses a cysteine point mutation in an unstructured loop of the PilA pilin subunit to label the pili through maleimide-based click chemistry. We first labeled the pili of untreated cells and then imaged TFP dynamics after a brief (5 min) exposure to MHQ or PBS control. Following a 5 min mock treatment with PBS, we saw TFP extending and retracting from 77% of the cells (n = 473) (Fig. 4, *G* and *H* and Movie S1). In contrast, when cells were treated with 2 mM MHQ for 5 min, only 4% (n = 493) of the cells exhibited any TFP extension or retraction events (Fig. 4, *G* and *H* and Movie S2). Since the TFP was labeled before MHQ treatment and the treatment was brief, this effect cannot be attributed to effects of MHQ on TFP expression or labeling. Thus, we conclude that MHQ inhibits TFP activity.

## Discussion

Here we sought to disrupt the early stages of single-cell surface attachment as a previously unexplored approach to combatting *P. aeruginosa*. We found that *P. aeruginosa* itself produces dispersal agents present in conditioned media. There are a variety of high-cell-density environments where a dispersal agent could be beneficial. For example, in a competition for colonizing a limited surface area, it might be beneficial to prioritize colonization by cells that have managed to grow to higher density. Other possibilities include dispersal helping the population respond to conditions of stress by promoting migration away from an undesirable surface or disrupting the surface attachment of competing bacterial species. Future studies will be required to determine if there are physiological triggers of dispersal or specific environments in which dispersal proves beneficial.

Our findings establish MHQ as a small molecule factor that is both produced at a low concentration by *P. aeruginosa* and sufficient to disperse surface-attached *P. aeruginosa* at high concentrations. While MHQ was previously shown to be present in *P. aeruginosa* cells (30), our study represents the first characterization of any biological function or synthesis pathway for this compound. Specifically, we show that MHQ is synthesized by the *pqsABCDE* operon that is also responsible for the biosynthesis of similar alkyl quinolones like HHQ. Whether MHQ is a by-product of making HHQ or an intentional product, its characterization presents an opportunity to learn about new ways for dispersing *P. aeruginosa*. Since MHQ was not sufficient to explain the dispersal activity of WT *P. aeruginosa* conditioned media, we are continuing to look for additional factors that work either alone or in conjunction with MHQ to induce dispersal. This work highlights the rich diversity of bioactive secondary metabolites produced by *P. aeruginosa* that have yet to be fully explored and characterized.

Characterizing its mechanism led to the surprising discovery that MHQ inhibits TFP dynamics. TFP inhibition appears to be sufficient to explain MHQ's activity as MHQ also inhibits TFP-dependent twitching motility and vertical surface attachment. Furthermore, mutants lacking TFP phenocopy MHQ with respect to surface attachment orientation and do not respond to MHQ in a twitching assay. Finally, while MHQ does not affect membrane permeability or polarization, polarization is necessary for TFP activity, and CCCP, a known depolarizer, was the only antibiotic capable of producing even mild dispersal. Others have proposed that disrupting ion channels can inhibit pilus activity (31), but MHQ does not depolarize bacteria. Thus, MHQ may reveal a new, polarization-independent way to disrupt TFP and TFP-mediated behaviors. While others have shown that TFP could be disrupted by a 30 min treatment with their compounds (32), we have shown that a 5 min treatment with MHQ leads to TFP disruption. TFP activity is implicated in multiple aspects of *P. aeruginosa* colonization and virulence (10, 33), such that these results suggest that MHQ may be useful for affecting other TFP-dependent behaviors beyond early surface attachment. In future studies, it will prove interesting to determine the specific mechanism by which MHQ disrupts the complex dynamical system of TFP activity (13).

MHQ has exciting potential applications for both the clinical and lab settings as a small molecule agent that rapidly disperses cells from a surface. MHQ could serve as a therapeutic in the treatment of deadly surface associated pathogens such as *P. aeruginosa.* As biofilms are notoriously difficult to treat with conventional antibiotics (34, 35, 36, 37, 38), MHQ could be particularly helpful for combatting the initial formation of biofilms, by removing cells from the surface before biofilms can become established. In the future, it will also be important to synthesize and characterize MHQ analogs to see if a derivative might increase its potency, as the concentrations currently required for its activity (EC_50_ = 1 mM) may be prohibitively high for some applications. Another potential hurdle is that MHQ's chemical similarity to other alkyl quinolones like HHQ could result in derivatives that induce signaling cross talk or cause cytotoxicity. We already ruled out some alkyl quinolone activities such as antibiotic activity and membrane depolarization but establishing the effect of MHQ on other activities such as quorum sensing will require further investigation. In any event, our work provides proof of principle for future efforts to identify and characterize small molecules that disperse bacteria and disrupt TFP activity.

## Experimental procedures

### Strains and growth conditions

A list of the strains can be found in Table 2. We used *P. aeruginosa* PA14 for the wild-type strain throughout this study. All strains were grown at 37 °C in liquid LB Miller (Difco) on a roller drum at 90 rpm.

**Table 2:** Bacterial strains

Strain description	Unique identifier	Reference
*P. aeruginosa strain UCBPP PA14*	ZG 38	Lab stock
PA14 *ΔpilA*	ZG 1713	This study
PA14 *ΔpqsABCDE*	ZG 1714	(12)
PA14 *ΔpqsR*	ZG 1738	Lab stock
PA14 *pqsA::MAR2xT7*	23,621	(20)
PA14 *pqsB::MAR2xT7*	42,596	(20)
PA14 *pqsC::MAR2xT7*	32,423	(20)
PA14 *pqsE::MAR2xT7*	45,262	(20)
PA14 *pqsH::MAR2xT7*	47,950	(20)
PA14 *pilA-T51 C*	ZG 1715	This study
*E. coli lptD 4213*	ZG 1598	Lab stock

### Strain construction

The *pilA*-Cys knock-in mutant were generated using two-step allelic exchange (13, 39). Briefly, the cloning vectors were created by digesting the pEXG2 backbone with the HindIII HF restriction enzyme (NEB). Knock-in vectors were created by amplifying the 500 bp flaking regions upstream and downstream of the mutation site using primers pilA-T51CC_P1/2/3/4 (Table 3). The overlapping primers pilA-T51C_P2/3 were chosen as reverse complement containing the point mutation. Both flanks were then joined using SOEing PCR with the flanking primers. The *pilA*-Cys construct was then ligated into the pEXG2 backbone using T4 DNA ligase (NEB). Next, the cloning vectors were electroporated into *E. coli* and the correct mutation was confirmed using PCR and Sanger sequencing with primers pEXG2_Ver1/2. For mating, 1.5 ml *E. coli* containing the vector were grown to OD 0.5. The *P. aeruginosa* recipient strain was grown overnight, and 0.5 ml culture was diluted 1:2 into fresh LB and incubated for 3 h at 42 °C. Both cultures were concentrated into 100 μl and spotted onto an LB agar plate and incubated overnight at 30 °C. The puddle was scraped off, resuspended into 150 μl PBS, spread onto a Vogel Bronner Minimal Medium plate containing 30 mg/ml gentamycin, and incubated 24 h at 37 °C. Six single colonies from the Vogel Bronner Minimal Medium plate were struck onto a 15% sucrose, no salt LB plate and incubated for 24 h at 30 °C. Several single colonies from the sucrose plate were screened for the correct mutation using PCR amplification with the flanking primers and Sanger sequencing.

**Table 3:** Primers

Primer name	Primer sequence	Usage
pilA-1	GATACAAAGCTTCTTGTTGCGCTGGGCCTG	*pilA* deletion
pilA-2	GGTACCTGCAGTCAGGGCCGCAACCACGATCATCAGTTC	*pilA* deletion
pilA-3	GAACTGATGATCGTGGTTGCGGCCCTGACTGCAGGTACC	*pilA* deletion
pilA-4	GATACAAAGCTTCATGAACAAGAGCAAGCGGC	*pilA* deletion
pilA-T51CC_P1	GATACAAAGCTTCCGCTGAGTTGAATTGTGTCG	*pilA* cysteine replacement
pilA-T51CC_P2	CTGGCTGCCAGCGCCAAGTGTCTTATTGGCGATAGCTCT GCC	*pilA* cysteine replacement
pilA-T51CC_P3	GGCAGAGCTATCGCCAATAAGACACTTGGCGCTGGCAG CCAG	*pilA* cysteine replacement
pilA-T51CC_P4	GATACAAAGCTTCCACCACAAACAGATGATTGCC	*pilA* cysteine replacement
pEXG2_Ver1	GTTGCATGGGCATAAAGTTGCC	Confirming inserts
pEXG2_Ver2	CGGGTCCTCAACGACAGG	Confirming inserts
PA14pilA_Seq1	GGCTGTTCAGGTCGCAGTAGG	Confirming exconjugants

The *pilA* deletion was constructed by two-step allelic exchange using plasmid pEXG2. Fragments directly upstream and downstream of the *pilA* gene were amplified from PA14 gDNA using primer pairs (pilA-1, pilA-2) and (pilA-3, pilA-4) (Table 3), respectively. Upstream and downstream fragments were fused together using overlap-extension PCR with primer pair (pilA-1, pilA-4), and the resulting fragment was cloned into the HindIII site of plasmid pEXG2. The pEXG2 plasmid was integrated into *P. aeruginosa* PA14 through conjugation with the donor strain *E. coli* S17. Exconjugants were selected on 30 μg/ml gentamycin LB plates, and then the mutants of interest were counter-selected on 5% sucrose LB plates.

### Microscopy

#### DISPEL assay

For the DISPEL assay, cells were grown to mid-log (OD_600_ = 0.6–0.8) unless otherwise noted (Fig. 1*C*) and 50 μl of the culture was added to a well of a 96-well plate (Corning 3904). After 10 min of attachment, the unattached cell cultures were aspirated off slowly. Wells were then incubated for 10 min with 50 μl of treatment. After incubation, treatment was aspirated off slowly and the wells were washed gently once with 50 μl 1× PBS, and then a final 50 μl of 1× PBS was added for imaging purposes. Wells were imagined on a Nikon TE2000 using a Nikon S Plan Fluor ELWD 20X/0.45 OFN22 PH1, Andor iXon DV-897, and μmanager 2.0 imaging software (40).

#### Orientation of individual cells

For the individual cell orientation experiments, cells were grown to mid-log (OD_600_ = 0.6–0.8) and a 50 μl droplet was placed on a 60 × 22 mm glass cover slip. After 5 min of attachment, treatment was added to the cells and incubated for 5 min. Cells were imaged using a Nikon Ti microscope using a Plan Apo λ 40X PH2, Hamamatsu Orca Flash 4, and Nikon NIS Elements imaging software.

#### Pilus labeling

Pili were labeled as described previously (13, 28, 29) with some modifications. Cells were grown to mid-log growth phase with an OD_600_ of 0.6 and 1 ml of culture was centrifuged at 8000*g* for 1 min. The culture supernatant was removed and cell pellets were resuspended in 50 μl of the removed supernatant to concentrate the cells. Concentrated cell suspensions were incubated with 0.5 μl of 0.5 mg/ml stock AF488-malemide (ThermoFisher) for 30 min at room temperature. Cells were centrifuged at 8000*g* for 1 min, the supernatant was removed, and 50 μl of the original supernatant was gently added and removed to wash the cells with minimal disturbance to the pellet. The cell pellet was then resuspended in 20–50 μl of the original culture supernatant. One microliter of labeled cells was added to a 60 × 22 mm glass coverslip. These cells were then treated with either 1× PBS as a control or 2 mM MHQ and incubated for 5 min at room temperature. After incubation, a 1% agarose (Invitrogen) PBS pad was used to sandwich cells between the coverslip and pad. One percent agarose PBS pads were made with 2 mM MHQ for imaging MHQ-treated cells. Cells were imaged using a Nikon Ti microscope using a Plan Apo λ 100X Oil PH3, GFPHQ Filter Cube (Ex:455–485, Em:500–545), Hamamatsu Orca Flash 4, and Nikon NIS Elements imaging software.

### Twitching assay

Colonies of cells were picked with a 10 μl pipette tip and stabbed through the agar of a LB 1.5% agar plate and placed at 30 °C for 4 days. After 4 days, the agar was gently removed from the dish and a sufficient volume of 0.5% (w/v) crystal violet in water was added to the plate until the surface covered. After 5 min of staining, the crystal violet solution was removed, and the plate was washed three times with water. The resulting crystal violet stained twitch rings were imaged using a Canon EOS Rebel T1i and measured in FIJI (41, 42, 43).

### Flow cytometry

Flow cytometry protocol was adapted from Martin *et al*. (44). In brief, overnight *E. coli lptD4213* and *P. aeruginosa* PA14 monocultures were grown to early-mid exponential phase (OD600 = 0.4–0.6) at 37 °C. Each culture was then diluted 1:10 into 1× PBS and treated with 2 mM MHQ, 5 uM CCCP, or 4 μg/ml polymyxin for 10 min. Cells were stained with the BacLight Bacterial Membrane Potential kit (ThermoFisher B34950). This kit uses DiOC2(3) to measure a cell's membrane potential as a ratio of green (488 nm ex, 525/50 nm em) to red (488 nm ex, 610/20 nm em) (45). Membrane integrity was measured by staining cells with TO-PRO-3, a dye that is excluded from cells with an intact membrane (640 nm ex, 670/30 nm em). The LSRII flow cytometer (BD Biosciences) at the Flow Cytometry Resource Facility, Princeton University, was used to measure the fluorescent intensities of both dyes in response to antibiotic or MHQ treatment. In total, 100,000 events were recorded for each data file. Data was analyzed using FlowJo v10 software (FlowJo LLC).

### Growth curves

Mid-log (OD_600_ = 0.6–0.8) *P. aeruginosa* cells were treated with antibiotics and MHQ (Novobiocin—10 mg/ml, Tetracycline—16 μg/ml, Trimethoprim—125 μg/ml, Gentamicin—6 μg/ml, CCCP—200 μM, MHQ—2 mM) for 10 min. Cells were spun and resuspend in equal volumes of drug-free LB twice. Cells were diluted 1:100 and grown for 10 h in a microplate reader (Tecan) at 37 °C with shaking.

### Image analysis

#### DISPEL assay

Images were analyzed using custom MATLAB (MathWorks) scripts to count the number of cells in the image. The data were normalized using the control images of PBS-treated or LB-treated cells. Activity was defined as

(1) $$\text{Activity}\;=\;1\;-\;\frac{\text{Average \# of cells in treatment well}}{\text{Average \# of cells in control well}}$$

Data was fit based on using a modified Hill equation

(2) $$y\;=\;a*\frac{x^{n}}{E{C_{50}}^{n}\;+\;x^{n}}\;+\;b$$

with *y* being the activity and *x* being the condition varied in the experiment. *a* and *b* sum to 1 and are the relative magnitude of the experimental variant and the no treatment offset, respectively. *EC*_*50*_ is the effective concentration at which the activity is 50% of the total effect. The cooperativity coefficient, *n*, refers the steepness of the transition between effect and no effect. For our data *n* was around 15.

#### Orientation of individual cells

Individual cells were hand-scored for their orientation to the surface as either vertical (on their pole) or horizontal (on their side). For each of three biological replicates, an image containing 250–1000 cells was scored. The fraction of cells vertical was calculated by dividing the number of cells vertical by the total number of cells scored. The average and standard deviation across biological replicates are shown (Fig. 4*B*).

#### Pilus activity

Individual cells were hand-scored for whether there was a pilus event within a 5-min movie. A pilus event was generously defined as any extension or retraction event regardless if the cycle was fully completed during the movie. For each of three biological replicates, an image containing 50–250 cells was scored. The fraction of cells with pilus activity was calculated by dividing the number of cells with a pilus event by the total number of cells scored. The average and standard deviation across biological replicates are shown (Fig. 4*F*).

### Large-scale culture and fractionation

For the large-scale culturing and purification of MHQ, 1 ml overnight culture was used as an inoculum for each 500 ml LB media batch and cultivated overnight at 37 °C shaken at 200 rpm. A total of 50 L total was generated. Two batches at a time were combined and centrifuged at 15,000*g* for 15 min (JLA 9.1000, Avanti JXN-30, 25 °C) to separate cells from the conditioned media. The conditioned media was shaken with Diaion HP-20 (Sigma-Aldrich), 50 ml for each 1 L combined batch of conditioned media, for 1 h and resins were eluted with MeOH. Subsequently, the extracts from all batches were combined and dried *in vacuo*. The total extract was resuspended with 500 ml H_2_O, then partitioned with 500 ml of ethyl acetate 15 times. The organic layers from each partition were combined and dried *in vacuo*.

The dried crude extract was resuspended in 5 ml MeOH and subjected to open-column chromatography by using Mega Bond Elut-C18, 70 g, 150 ml (Agilent Technologies) with stepwise elution with MeCN: H_2_O (10:90, 20:80, 20:80, 30:70, 30:70, 50:50) and a final wash 100% MeOH. At this point, and at each subsequent step of the purification process, a portion of each fraction was dried *in vacuo*, resuspended in 1x PBS, and used in the DISPEL assay to determine activity (Fig. 2, *A* and *B* and DISPEL assay).

Active fraction was further purified on another Mega Bond Elut-C18, 10 ml (Agilent Technologies) with stepwise elution with MeCN: H_2_O (10:90, 50:50, 100:0) and a final wash 100% MeOH.

Active fraction was purified by HPLC on a C18 reverse-phase column (Poroshell 120 EC-C18, 9.4 × 250 mm, 4 μm) with gradient 0–15 min: 30–100 % A, 15–21 min: 100 % A, 21–21.5 min: 100–30 % A, at a flow rate of 1.25 ml/min. (Solvent A: MeCN with 0.01% TFA; B: H_2_O with 0.01% TFA).

The active fraction was further purified by HPLC on a C18 reverse-phase column (Poroshell 120 EC-C18, 9.4 × 250 mm, 4 μm) with gradient 0–12 min: 20–60 % A, 12–12.1 min: 100 % A, 12.1–16.8 min: 100 % A, 16.8–17 min: 100–20 % A, at a flow rate of 1.25 ml/min. (Solvent A: MeCN with 0.01% TFA; B: H_2_O with 0.01% TFA).

The active fraction was further purified by HPLC on a C18 reverse-phase column (Poroshell 120 EC-C18, 9.4 × 250 mm, 4 μm) with isocratic elution 0–23 min: 15 % A, at a flow rate of 1.3 ml/min. (Solvent A: MeCN with 0.01% TFA; B: H_2_O with 0.01% TFA).

The active fraction was further purified by HPLC on a C18 reverse-phase column (Poroshell 120 EC-C18, 4.6 × 250 mm, 4 μm) with isocratic elution 0–17 min: 17 % A, at a flow rate of 0.6 ml/min. (Solvent A: MeCN with 0.01% TFA; B: H_2_O with 0.01% TFA). Finally, 1.3 mg (0.09 mg/L) of final fraction was obtained (VI, Fig. 2*B*).

### Structural elucidation of MHQ

VI was isolated as a slightly orange solid. HPLC-HRMS (using a Shimadzu HPLC and Thermo LTQ Orbitrap XL MS) established that the majority of VI corresponded to the *m/z* = 160.07559 [M + H]^+^, with a predicted molecular formula C_10_H_9_NO (calculated m/z = 160.0762). ^1^H-NMR spectrum of VI indicated one hydroxyl, four aromatic, one methine, three methyl protons (Table 1). In the ^13^C-NMR spectrum, ten unique signals were observed, including nine aromatic carbons, one methyl (Table 1). The bicyclic structure of 2-methyl-4-hydroxyquinoline (MHQ) seen in Figure 2*E* was elucidated by COSY and HMBC experiments (Figs. S1–S3). The structure of MHQ was further confirmed by comparison with MS, ^1^H-NMR and ^13^C-NMR spectra for a commercially available authentic standard of the same molecule (Sigma H43806) as well as ^1^H-NMR data for MHQ from a previous reference reporting the same molecule (Fig. S3 and Table 1) (30).

### HPLC-MS curve information

For both relative and absolute quantification of MHQ in conditioned media and VI, we used HPLC-MS (Agilent Single Quad) using a C18 reverse-phase column (Poroshell 120 EC-C18, 4.6 × 100 mm, 2.7 μm). For standard addition, the desired concentration of MHQ was added to the sample and then run on the HPLC-MS. MHQ ion count was determined by integrating the peak of the extracted ion chromatogram for 160.1. The absolute concentration of MHQ was calculated for the conditioned media by taking the intercept of the linear fit for the standard curve in conditioned media.

## Data availability

The data supporting the findings of the study are available in this article and its Supplementary Information Files. Additionally, the raw data that support the findings of this study are available from the corresponding author upon request.

## Conflict of interest

A provisional patent describing MHQ as a potential therapeutic in surface dispersal has been filed.

## References

[1] Santajit S., Indrawattana N. (2016). Mechanisms of antimicrobial resistance in ESKAPE pathogens. Biomed. Res. Int..

[2] Magill S.S., Edwards J.R., Bamberg W., Beldavs Z.G., Dumyati G., Kainer M.A., Lynfield R., Maloney M., Mcallister-Hollod L., Nadle J., Ray S.M., Thompson D.L., Wilson L.E., Fridkin S.K. (2014). Multistate point-prevalence survey of health care–associated infections for the emerging infections program healthcare-associated infections and antimicrobial use prevalence survey team. N. Engl. J. Med..

[3] Hall S., Mcdermott C., Anoopkumar-Dukie S., Mcfarland A.J., Forbes A., Perkins A.V., Davey A.K., Chess-Williams R., Kiefel M.J., Arora D., Grant G.D. (2016). Cellular effects of pyocyanin, a secreted virulence factor of Pseudomonas aeruginosa. Toxins (Basel).

[4] Soberón-Chávez G., Lépine F., Déziel E. (2005). Production of rhamnolipids by Pseudomonas aeruginosa. Appl. Microbiol. Biotechnol..

[5] Morihara K., Tsuzuki H., Oka T., Inoue H., Ebata M. (1965). Pseudomonas aeruginosa elastase isolation, crystallization, and preliminary characterization. J. Biol. Chem..

[6] Kaletsky R., Moore R.S., Vrla G.D., Parsons L.R., Gitai Z., Murphy C.T. (2020). C. elegans interprets bacterial non-coding RNAs to learn pathogenic avoidance. Nature.

[7] Lee D.G., Urbach J.M., Wu G., Liberati N.T., Feinbaum R.L., Miyata S., Diggins L.T., He J., Saucier M., Déziel E., Friedman L., Li L., Grills G., Montgomery K., Kucherlapati R. (2006). Genomic analysis reveals that Pseudomonas aeruginosa virulence is combinatorial. Genome Biol..

[8] Feinbaum R.L., Urbach J.M., Liberati N.T., Djonovic S., Adonizio A., Carvunis A.-R., Ausubel F.M. (2012). Genome-wide identification of Pseudomonas aeruginosa virulence-related genes using a Caenorhabditis elegans infection model. PLoS Pathog..

[9] Siryaporn A., Kuchma S.L., O 'toole G.A., Gitai Z. (2014). Surface attachment induces Pseudomonas aeruginosa virulence. Proc. Natl. Acad. Sci. U. S. A..

[10] Persat A., Inclan Y.F., Engel J.N., Stone H.A., Gitai Z., Harwood C.S. (2015). Type IV pili mechanochemically regulate virulence factors in Pseudomonas aeruginosa. Proc. Natl. Acad. Sci. U. S. A..

[11] Lee C.K., de Anda J., Baker A.E., Bennett R.R., Luo Y., Lee E.Y., Keefe J.A., Helali J.S., Ma J., Zhao K., Golestanian R., O'Toole G.A., Wong G.C.L. (2018). Multigenerational memory and adaptive adhesion in early bacterial biofilm communities. Proc. Natl. Acad. Sci. U. S. A..

[12] Chuang S.K., Vrla G.D., Fröhlich K.S., Gitai Z. (2019). Surface association sensitizes Pseudomonas aeruginosa to quorum sensing. Nat. Commun..

[13] Koch M.D., Fei C., Wingreen N.S., Shaevitz J.W., Gitai Z. (2020). Competitive binding of independent extension and retraction motors explains the quantitative dynamics of type IV pili. bioRxiv.

[14] Huang W., Brewer L.K., Jones J.W., Nguyen A.T., Marcu A., Wishart D.S., Oglesby-Sherrouse A.G., Kane M.A., Wilks A. (2017). PAMDB: A comprehensive Pseudomonas aeruginosa metabolome database. Nucleic Acids Res..

[15] Davies D.G., Marques C.N.H. (2009). A fatty acid messenger is responsible for inducing dispersion in microbial biofilms. J. Bacteriol..

[16] Leiman S.A., May J.M., Lebar M.D., Kahne D., Kolter R., Losick R. (2013). D-Amino acids indirectly inhibit biofilm formation in Bacillus subtilis by interfering with protein synthesis. J. Bacteriol..

[17] Petrova O.E., Sauer K. (2012). Dispersion by Pseudomonas aeruginosa requires an unusual posttranslational modification of BdlA. Proc. Natl. Acad. Sci. U. S. A..

[18] Barraud N., Hassett D.J., Hwang S.-H., Rice S.A., Kjelleberg S., Webb J.S. (2006). Involvement of nitric oxide in biofilm dispersal of Pseudomonas aeruginosa. J. Bacteriol..

[19] Kang O.Y., Park S.J., Ahn H., Jeong K.C., Lim H.J. (2019). Structural assignment of the enol-keto tautomers of one-pot synthesized 4-hydroxyquinolines/4-quinolones. Org. Chem. Front..

[20] Liberati N.T., Urbach J.M., Miyata S., Lee D.G., Drenkard E., Wu G., Villanueva J., Wei T., Ausubel F.M. (2006). An ordered, nonredundant library of Pseudomonas aeruginosa strain PA14 transposon insertion mutants. Proc. Natl. Acad. Sci. U. S. A..

[21] Drees S.L., Fetzner S. (2015). PqsE of Pseudomonas aeruginosa acts as pathway-specific thioesterase in the biosynthesis of alkylquinolone signaling molecules. Chem. Biol..

[22] Lin J., Cheng J., Wang Y., Shen X. (2018). The Pseudomonas quinolone signal (PQS): Not just for quorum sensing anymore. Front. Cell Infect. Microbiol..

[23] Dubern J.F., Diggle S.P. (2008). Quorum sensing by 2-alkyl-4-quinolones in Pseudomonas aeruginosa and other bacterial species. Mol. BioSystems.

[24] Heeb S., Fletcher M.P., Chhabra S.R., Diggle S.P., Williams P., Cámara M. (2011). Quinolones: From antibiotics to autoinducers. FEMS Microbiol. Rev..

[25] Conrad J.C., Gibiansky M.L., Jin F., Gordon V.D., Motto D.A., Mathewson M.A., Stopka W.G., Zelasko D.C., Shrout J.D., Wong G.C.L. (2011). Flagella and pili-mediated near-surface single-cell motility mechanisms in P. aeruginosa. Biophys. J..

[26] de Anda J., Lee E.Y., Lee C.K., Bennett R.R., Ji X., Soltani S., Harrison M.C., Baker A.E., Luo Y., Chou T., O'Toole G.A., Armani A.M., Golestanian R., Wong G.C.L. (2017). High-speed 4D computational microscopy of bacterial surface motility. ACS Nano..

[27] Lee C.K., Vachier J., de Anda J., Zhao K., Baker A.E., Bennett R.R., Armbruster C.R., Lewis K.A., Tarnopol R.L., Lomba C.J., Hogan D.A., Parsek M.R., O'toole G.A., Golestanian R., Wong G.C.L. (2020). Social cooperativity of bacteria during reversible surface attachment in young biofilms: A quantitative comparison of Pseudomonas aeruginosa PA14 and PAO1. mBio..

[28] Ellison C.K., Kan J., Dillard R.S., Kysela D.T., Ducret A., Berne C., Hampton C.M., Ke Z., Wright E.R., Biais N., Dalia A.B., Brun Y.v. (2017). Obstruction of pilus retraction stimulates bacterial surface sensing. Science.

[29] Ellison C.K., Dalia T.N., Dalia A.B., Brun Y.V. (2019). Real-time microscopy and physical perturbation of bacterial pili using maleimide-conjugated molecules. Nat. Protoc..

[30] Royt P.W., Honeychuck R.v., Pant R.R., Rogers M.L., Asher L.v., Lloyd J.R., Carlos W.E., Belkin H.E., Patwardhan S. (2007). Iron- and 4-hydroxy-2-alkylquinoline-containing periplasmic inclusion bodies of Pseudomonas aeruginosa: A chemical analysis. Bioorg. Chem..

[31] Denis K., le Bris M., le Guennec L., Barnier J.P., Faure C., Gouge A., Bouzinba-Ségard H., Jamet A., Euphrasie D., Durel B., Barois N., Pelissier P., Morand P.C., Coureuil M., Lafont F. (2019). Targeting Type IV pili as an antivirulence strategy against invasive meningococcal disease. Nat. Microbiol..

[32] Aubey F., Corre J.P., Kong Y., Xu X., Obino D., Goussard S., Lapeyrere C., Souphron J., Couturier C., Renard S., Duménil G. (2019). Inhibitors of the Neisseria meningitidis PilF ATPase provoke type IV pilus disassembly. Proc. Natl. Acad. Sci. U. S. A..

[33] O'Toole G.A., Kolter R. (1998). Flagellar and twitching motility are necessary for *Pseudomonas aeruginosa* biofilm development. Mol. Microbiol..

[34] Mulcahy L.R., Isabella V.M., Lewis K. (2014). Pseudomonas aeruginosa biofilms in disease. Microb. Ecol..

[35] Davies D. (2003). Understanding biofilm resistance to antibacterial agents. Nat. Rev..

[36] Suci P.A., Mittelman M.W., Yu F.P., Geesey G.G. (1994). Investigation of ciprofloxacin penetration into Pseudomonas-aeruginosa biofilms. Antimicrob. Agents Chemother..

[37] Epstein A.K., Pokroy B., Seminara A., Aizenberg J. (2011). Bacterial biofilm shows persistent resistance to liquid wetting and gas penetration. Proc. Natl. Acad. Sci. U. S. A..

[38] Mah T.F.C., O'Toole G.A. (2001). Mechanisms of biofilm resistance to antimicrobial agents. Trends Microbiol..

[39] Hmelo L.R., Borlee B.R., Almblad H., Love M.E., Randall T.E., Tseng B.S., Lin C., Irie Y., Storek K.M., Yang J.J., Siehnel R.J., Howell P.L., Singh P.K., Tolker-Nielsen T., Parsek M.R. (2015). Precision-engineering the Pseudomonas aeruginosa genome with two-step allelic exchange. Nat. Protoc..

[40] Edelstein A.D., Tsuchida M.A., Amodaj N., Pinkard H., Vale R.D., Stuurman N. (2014). Advanced methods of microscope control using μManager software. J. Biol. Methods.

[41] Schindelin J., Arganda-Carreras I., Frise E., Kaynig V., Longair M., Pietzsch T., Preibisch S., Rueden C., Saalfeld S., Schmid B., Tinevez J.Y., White D.J., Hartenstein V., Eliceiri K., Tomancak P. (2012). Fiji: An open-source platform for biological-image analysis. Nat. Methods.

[42] Rueden C.T., Schindelin J., Hiner M.C., DeZonia B.E., Walter A.E., Arena E.T., Eliceiri K.W. (2017). ImageJ2: ImageJ for the next generation of scientific image data. BMC Bioinformatics.

[43] Schneider C.A., Rasband W.S., Eliceiri K.W. (2012). NIH Image to ImageJ: 25 years of image analysis. Nat. Methods.

[44] Ii J.K.M., Sheehan J.P., Bratton B.P., Savitski M.M., Wilson M.Z., Correspondence G., Moore G.M., Mateus A., Li S.H.-J., Kim H., Rabinowitz J.D., Typas A., Gitai Z. (2020). A dual-mechanism antibiotic kills gram-negative bacteria and avoids drug resistance. Cell.

[45] Novo D., Perlmutter N.G., Hunt R.H., Shapiro H.M. (1999). Accurate flow cytometric membrane potential measurement in bacteria using diethyloxacarbocyanine and a ratiometric technique. Cytometry.

