# Supplementary material for: Pseudomonas aeruginosa detachment from surfaces via a self-made small molecule
Source: J Biol Chem. 2021 Jan 12;296:100279. doi: 10.1016/j.jbc.2021.100279 (PMC7949062; doi:10.1016/j.jbc.2021.100279)
Supplement: Figures S1 to S7 [file mmc1.pdf]

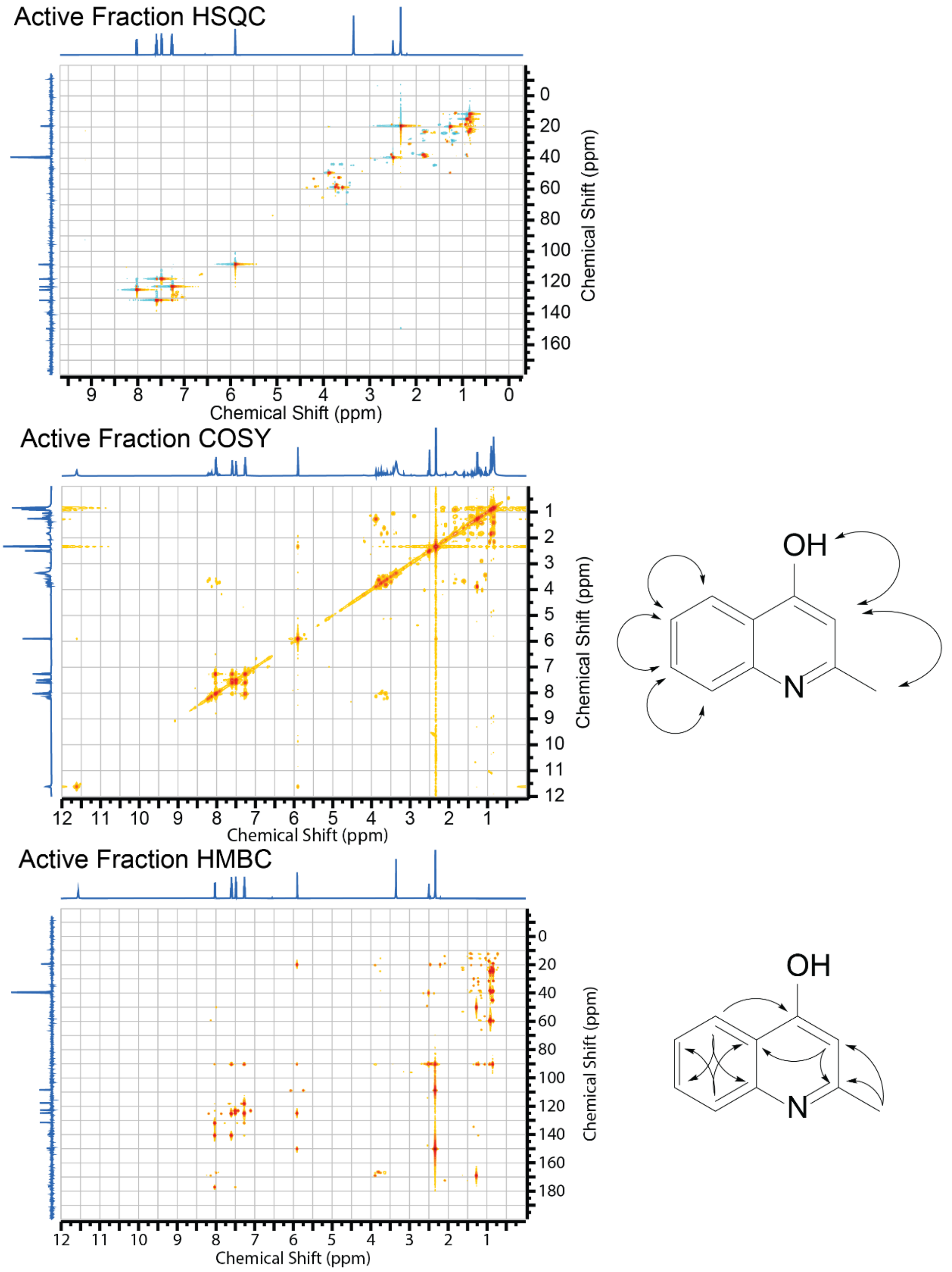

Fig. S1. 2D NMR spectra (HSQC, COSY, HMBC) of **VI** used to elucidate the structure of MHQ. All spectra were measured in DMSO-d6 at 295 K. The structure of MHQ is shown with selected correlations full arrows (COSY) and half arrows (HBMC).

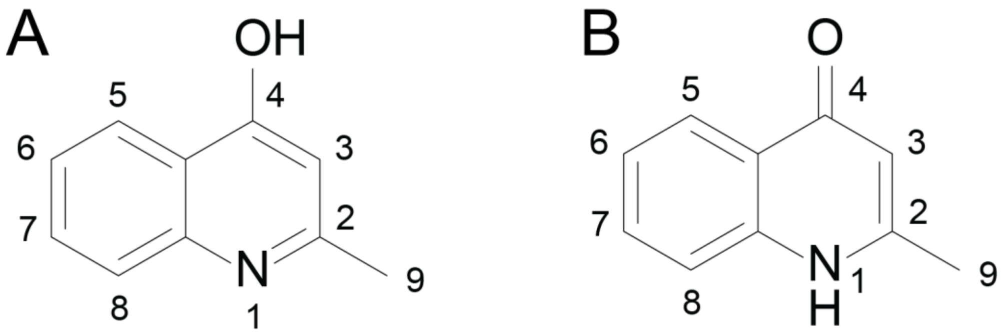

Fig. S2. Tautomers of MHQ shown in enol (A) and keto (B) forms. Nuclear numbering for peak assignment in Table S3.

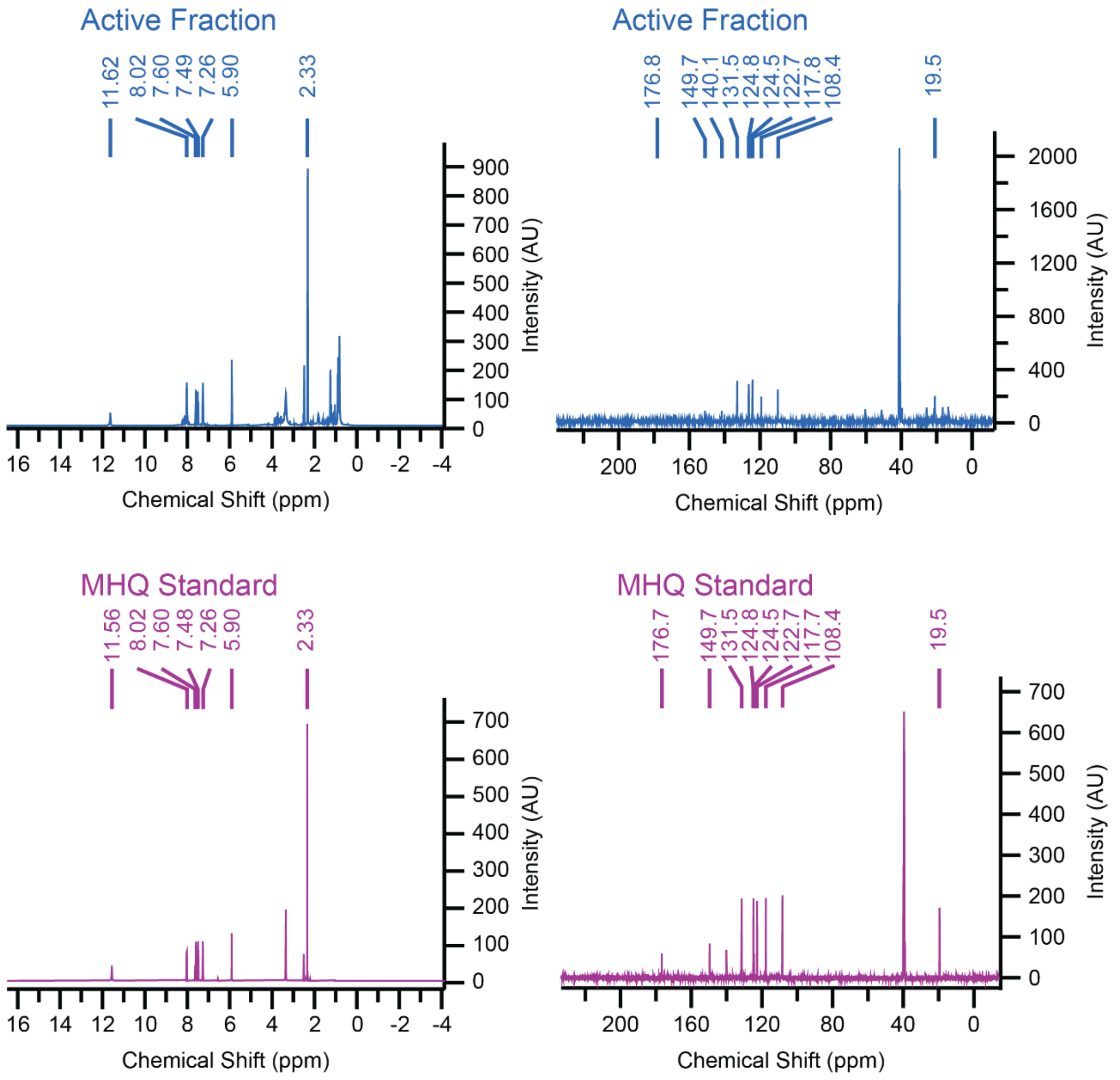

Fig. S3. 1D NMR spectra (^{1}H-NMR and ^{13}C-NMR) of **VI** and commercially available MHQ. All spectra were measured in DMSO-d6 at 295 K.

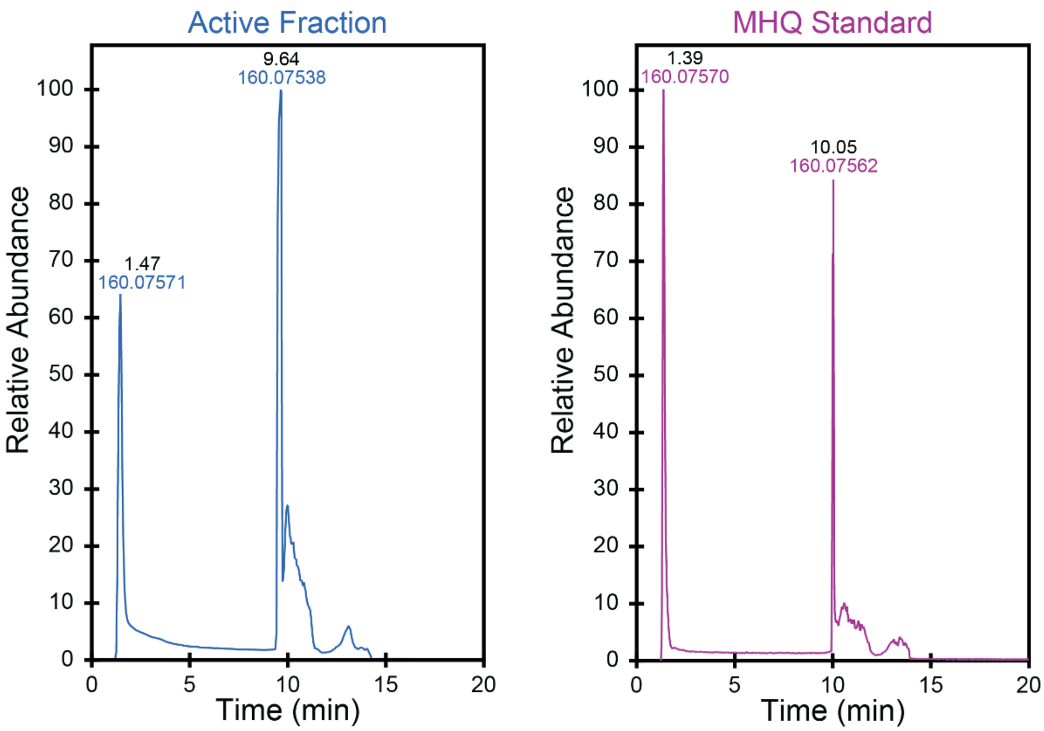

Fig. S4. HPLC-HRMS retention time traces of 160.07559 m/z in **VI** and commercially available MHQ.

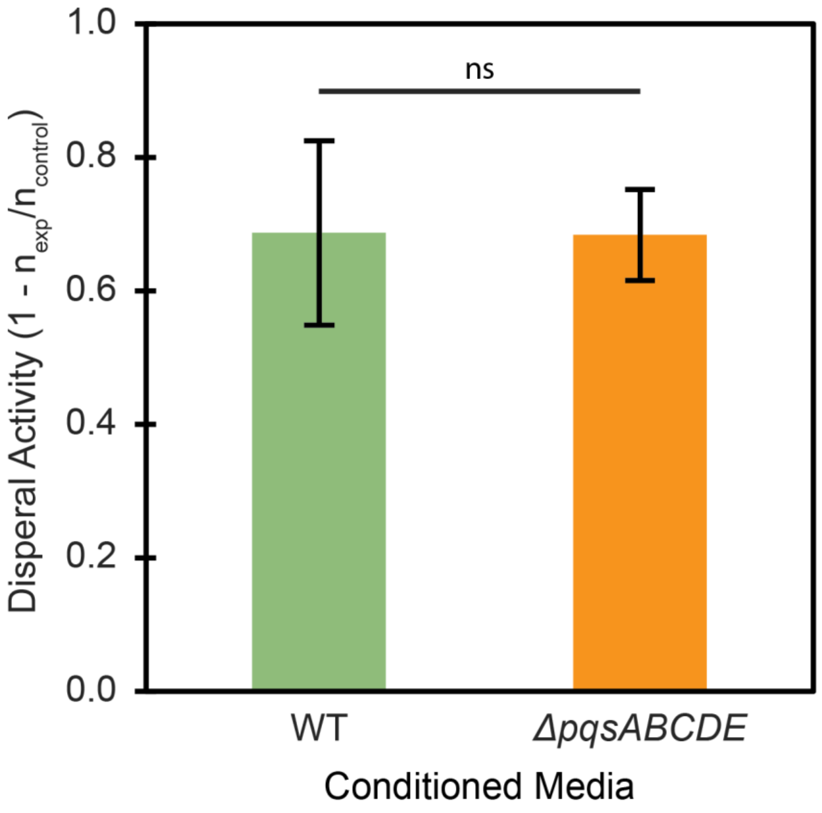

Fig. S5. Conditioned media from the *ΔpqsABCDE* has full dispersal activity. Mean and standard deviation shown from 5 biological replicates. ns p-value > 0.05 from Student's t-test**.**

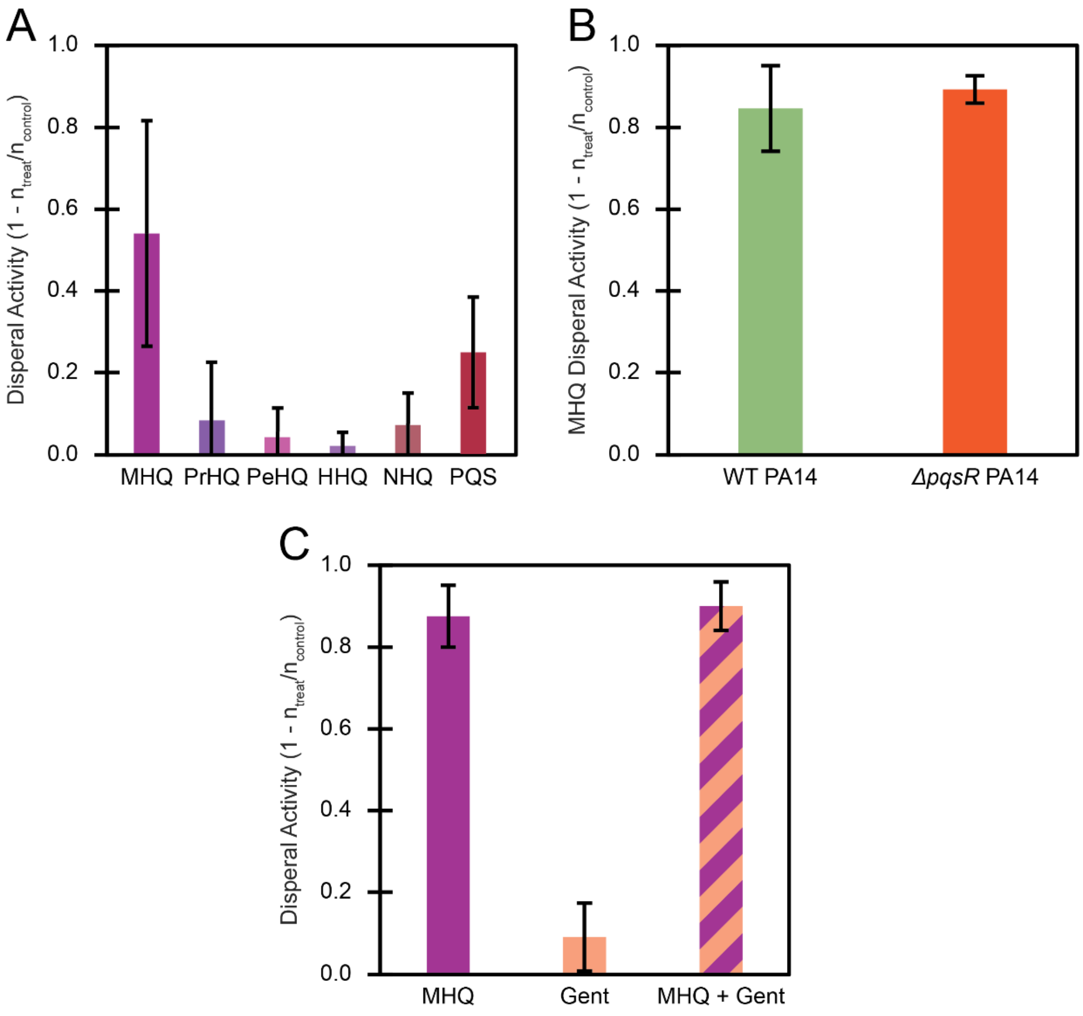

Fig. S6. MHQ is specific for dispersal and does not function through PqsR (A) Alkyl-quinolones other than MHQ do not cause dispersal activity in the DISPEL assay against mid-log (OD600 0.6-0.8) *P. aeruginosa* cells. Mean and standard deviation shown from 3 biological replicates. MHQ – 1 mM, Propyl-HQ – 1 mM, Pentyl-HQ – 20 µM, HHQ – 20 µM, Nonyl HQ – 20 µM, PQS – 20 µM. (B) Dispersal activity of 2 mM MHQ on mid-log (OD600 0.6-0.8) WT or *ΔpqsR P. aeruginosa* cells. Mean and standard deviation shown from 3 biological replicates. (C) Dispersal activity of 2 mM MHQ and 6 µg/mL gentamycin separate and in co-treatment on mid-

log (OD600 0.6-0.8) *P. aeruginosa* cells. Mean and standard deviation shown from 3 biological replicates.

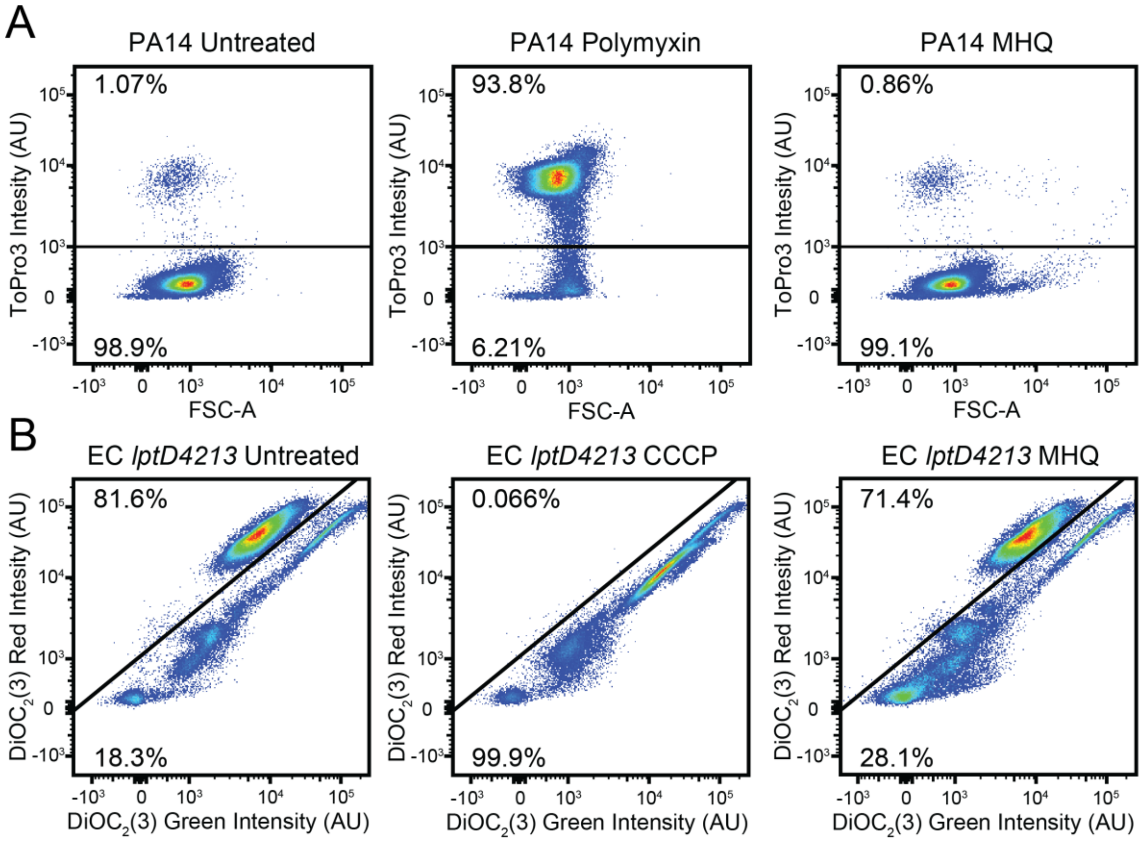

Fig. S7. Flow Cytometry analysis of membrane integrity and depolarization following MHQ treatment. (A) *P. aeruginosa* cells were stained with ToPro3 following either no treatment, polymyxin treatment, or MHQ treatment. Increased ToPro3 staining is indicative of cell permeabilization. (B) *E. coli lptD4213* cells were stained with DiOC2(3) following either no treatment, CCCP treatment, or MHQ treatment. Fluorescence shift towards green indicate membrane depolarization.

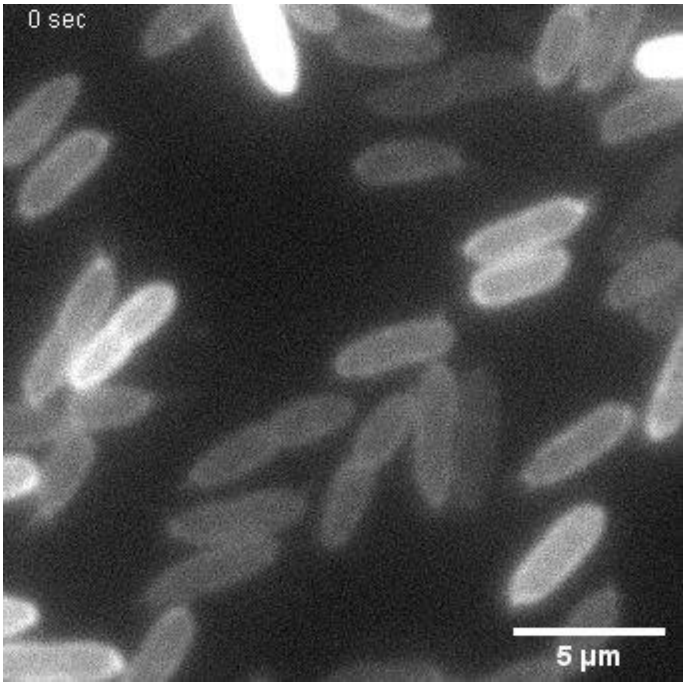

Movie S1 (separate file). Pilus activity of PBS Treated *P. aeruginosa* cells. Pili are fluorescently labeled. To account for photobleaching images were normalized to saturate 0.3% of pixels in each frame.

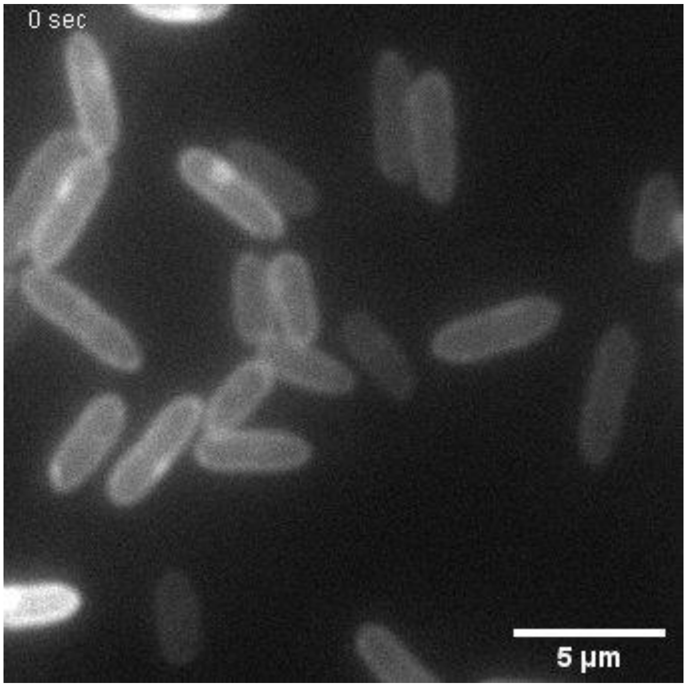

Movie S2 (separate file). Pilus activity of MHQ Treated *P. aeruginosa* cells. Pili are fluorescently labeled. To account for photobleaching images were normalized to saturate 0.3% of pixels in each frame.
